# Performance of a Tower-Shaped Integrated Ecological Purification Device for Pollutants Removal from Domestic Sewage in Rural Areas

**DOI:** 10.3390/ijerph192417014

**Published:** 2022-12-18

**Authors:** Min Yan, Jian Zhang, Xiaoguo Wang, Xin Lu

**Affiliations:** 1 College of Geography and Environmental Science, Northwest Normal University, Lanzhou 730070, China; 2 Institute of Mountain Hazards and Environment, Chinese Academy of Sciences, Chengdu 610041, China

**Keywords:** domestic sewage, tower-shaped integrated ecological purification device, removal efficiency, pollutants

## Abstract

With the continuous development of China’s modern economy and agricultural society, the discharge of rural sewage has been recognized as a major threat to the safety of the rural ecological environment. This study discussed the purification efficiency of a tower-shaped integrated ecological purification device (TIEPD)—consisting of a measuring tank, detention tank and three-stage purification unit—towards various common pollutants in rural areas during operation and tested the stability and efficiency of the TIEPD under different rural life events (fair activity days and nonfair activity days) and different precipitation intensities (light rain, moderate rain and heavy rain). The results showed that the average removal efficiencies of the TIEPD towards chemical oxygen demand, ammonia nitrogen, total nitrogen and total phosphorus were 69%, 67%, 54% and 73%, respectively. The average effluent concentration of each pollutant can meet the standard of the discharge of pollutants in China. The system exhibited good stability in removing pollutants and good ecological and economic benefits. This study provides the treatment of domestic sewage in the upper reaches of the Yangtze River and in mountainous areas of China and strengthens the prevention and control of rural nonpoint source pollution.

## 1. Introduction

With the continuous development of China’s modern economy and agricultural society, rural living standards are constantly improving. At the same time, the discharge of rural sewage has been recognized as a major threat to the safety of the rural ecological environment, and the methods for domestic sewage treatment in rural areas have become an emergency issue for the government [1,2,3]. In addition, water quality has deteriorated in many regions such as Europe [4], the USA [5], and South Korea [6]. At present, the construction of the rural collective economy in China is relatively backwards, the rural infrastructure is greatly behind cities and towns, and there is a lack of the necessary sewage treatment facilities and collection systems. Most of the sewage is not treated and is directly discharged into neighboring water bodies or spilled on the ground [7]. This has caused the eutrophication of the surrounding surface water in developing countries, and particularly in China [8,9,10]. The statistical findings from the First National Pollution Census Bulletin of China show that rural nonpoint source pollution (RNPS) accounts for approximately half of the total water pollution, 57.2% of the total nitrogen (TN) and 67.4% of the total phosphorus (TP) [11], seriously affecting water quality and posing a serious threat to the safety of human life and environmental sustainability [12,13,14]. As a result, identifying suitable methods of domestic sewage treatment for rural areas, particularly nitrogen and phosphorus removal, has been identified as a challenging management issue [7,15].

Centralized sewage treatment plants based on activated sludge or bacterial bed processes used in cities are not applicable in rural areas due to the dispersed rural population, as well as the professional skills and cost of constructing sewage collectors [16,17,18,19]. Thus, viable rural domestic sewage treatment technologies must be characterized by a simple design, low sludge production, treatment efficiency, simple maintenance and low operating costs [2,19,20,21]. At present, several solutions for treating domestic sewage effluent are already in operation in rural areas around the world, including the construction of wetlands, soil infiltration trenches, and vegetation-based sewage treatment [22,23,24,25]. However, these measures still have many shortcomings in the purification process of rural domestic sewage, such as effluent quality fluctuation, susceptibility to temperature, wetland plant species and pollutant concentration [26]. These measures would also be limited in areas where land demand is high, particularly in mountainous areas where there is less land available [1]. It is therefore a great challenge to purify domestic sewage in mountainous villages and towns through ecological engineering under the premise of a low economy and reduced land demand.

A tower-shaped integrated ecological purification device (TIEPD) is a new type of combined rural sewage treatment process that uses gravity, biological energy, solar energy and other forms of ecological energy, makes full use of the natural terrain in mountainous areas, represents a cascade purification system, covers a small area, is not restricted by terrain, and is more suitable for rural and small towns. It can filter pollutants through various biogeochemical processes before runoff enters the downstream receiving system, thereby reducing the nutrient load of domestic sewage in rural areas [15,27]. In the TIEPD, plant absorption, biological substrate transformation, aeration, and strengthening medium adsorption are combined to form a multistage plant-microbe-animal integrated ecological purification treatment process, which is better than a single process [28]. Moreover, rural domestic sewage treatment needs to consider a variety of factors. For instance, precipitation intensity and rural life events would affect the load of rural sewage, thus affecting the removal efficiency of pollutants [28,29].

In the central, hilly areas of Sichuan, the arbitrary discharge of domestic sewage by residents has become an important source of local agricultural surface pollution and a major factor affecting the safety of the water environment, and the amount of sewage discharge varies significantly over time. According to the characteristics of domestic sewage, a TIEPD was used to treat rural domestic sewage in a typical mountainous village in Yanting County, Sichuan Province, China. Combined with the local conditions, water samples were analyzed under different rural life events (fair activity days and nonfair activity days) and different precipitation intensities (light rain, moderate rain and heavy rain). By analyzing the contents of different indices, such as total nitrogen (TN), total phosphorus (TP), ammonia nitrogen (AN) and chemical oxygen demand (COD) in water and the concentration after purification by the TIEPD, the treatment efficiency and removal characteristics of the TIEPD for rural domestic sewage were discussed, and the removal mechanism and influencing factors were explored. Specifically, the objective of this study was to evaluate the performance of the TIEPD in treating domestic sewage in a rural township and to analyze the capacity of the TIEPD to remove nutrients under different loading conditions. According to this study, a scientific basis is provided in order to relieve the pressure of domestic sewage treatment for rural residents in subtropical mountainous areas and strengthening the prevention and control of RNPS pollution.

## 2. Materials and Methods

### 2.1. Study Area

This study was conducted in a rural township in a catchment in the hilly area of central Sichuan Province near the Yanting Agro-ecological Experimental Station of Purple Soil, Chinese Academy of Sciences, Southwest China (105°27′24″ E, 31°16′31″ N) [30]. The region has a moderate subtropical monsoon climate with an annual mean temperature of 17.3 °C and an annual mean precipitation of 826 mm [15,31]. The precipitation intensity is concentrated between May and September. The soil type is purple soil with a loamy texture. The study area has approximately 500 inhabitants per km^2^. The village has many restaurants, tea rooms and shops. There is a large village fair with up to 3000 people every 2–3 days, and the streets are not cleaned after the fair. Every day, approximately 30–150 m^3^ of domestic wastewater is generated and discharged directly into the sewer in the village. Approximately 0.86–4.31 kg N d^−1^ and 0.05–0.23 kg P d^−1^ are produced daily [15]. Additionally, this township has no sewage treatment facility.

### 2.2. Experimental Design

**Figure 1:**
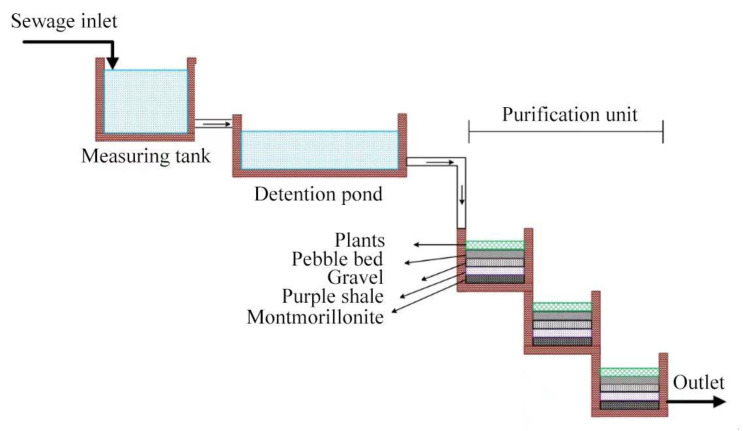
Flow chart of the sewage treatment of the tower-shaped integrated ecological purification device (TIEPD).

The main sources of domestic sewage in the town include kitchen sewage, washing sewage and flushing water. The sewage is discharged into the natural drainage ditch on both sides of the village road by the sewer pipe of each household (rain and sewage are not separated). This causes solid pollutants, demand pollutants, nutritional pollutants and other pollution. Therefore, a confluence ditch is built at a low-lying place at the end of the drainage ditch, which flows into the measuring tank by gravity (2 m × 2 m × 2 m), and the sewage discharge is measured by a thin-walled triangular weir (90°) [32]. The sewage from the measuring tank flows into the detention tank (4.5 m × 1.5 m × 1.5 m) to stabilize the sewage. The inlet of the detention tank is provided with a bamboo grill, with a spacing of 3 cm, and the water outlet is provided with a bamboo grill with a spacing of 0.5 cm. Then, the sewage enters the three-stage purification unit. The concrete ditches are constructed based on the elevation difference of the hill area. They are constructed by an amphibious plant reaction filter (1.5 m × 1.5 m × 1.5 m) containing emergent plants such as *Hydrocotyle verticillata* and *Ficus lacor* and submerged plants such as *Myriophyllum verticillatum L*. and connected from high to low. The combination of emergent and submerged plants increases the plant diversity and vegetation coverage and increases the pollutant absorption. The plant density transplanted in each stage was higher than 80%. In addition, there were animals in the reaction ponds such as amphibious animals, microorganisms and plankton. Plankton include protozoa, algae and some crustaceans and molluscs. Plankton directly ingest pollutants, microorganisms and plant secretions, sloughs, etc., to clean the environment of the reaction pond. Amphibious animals can accumulate pollutants to improve the permeability of the sediment medium layer, promote the absorption of pollutants by plant roots, and prevent the water outlet from being blocked. In addition to the absorption plants, oxidation-reduction microorganisms and ingestion plankton, the amphibious animals further enrich the food chains and improve the sewage treatment efficiency and self-purification function of the reaction pond. The gravity drop channel is set by using the elevation difference to form an oxidation-removal environment with an amphibious plant configuration and alternating dry and wet conditions. In the system tank, pebbles of 5–10 cm in size, gravel of 1–5 cm in size, purple shale of 0.5–2 mm in size and montmorillonite of 0.01–0.03 mm in size are laid, in order from high to low, with a thickness of 10–20 cm in each layer. They can filter pollutants, layer by layer, and form an upper aerobic-lower anaerobic compound environment and are provided with microbial strains for nitrogen (N) and phosphorus (P) removal. In the three-stage purification unit, the material and thickness of the filter layer laid in each stage were the same. The system tank is made of reinforced concrete and built at the foot of the slope, according to the terrain, to avoid occupying cultivated land. The construction cost is approximately 825 USD/m^2^. It takes 60 m^2^ to treat the sewage of the village, and the construction cost is much lower than that of ordinary domestic sewage treatment plants. The structure and process of the TIEPD are shown in Figure 1.

### 2.3. Sampling and Analysis

The performance of the TIEPD was monitored for one year (August 2018 to July 2019). In the study area, days ending with 2, 5 and 8 in each month were fair activity days, and the other days were nonfair activity days. Four to five fair activity days and four to five nonfair activity days were selected each month, and samples were taken at the entrance and exit, respectively, with three replicates at each sampling point. According to the discharge characteristics of rural domestic sewage, the samples were collected at three time periods of morning (7:00–8:00), noon (12:00–13:00) and evening (18:00–19:00), and the average value of the pollutants was used to represent the sewage characteristics of the sampling point on that day. In addition, the precipitation events in this year were divided into light rain (≤9.9 mm), moderate rain (10–24.9 mm) and heavy rain (>24.9 mm). During the rainy season (May to September), approximately 30 light, 15 moderate and 10 heavy rain samples were collected. Each sample included three repeated samples to test the operation effect of the TIEPD under different precipitation intensities.

The sample indices measured in this experiment included inlet and outlet water quality indices, including COD, AN, TN, TP and pH. The COD concentrations of the samples were determined using ultraviolet (UV)-visible (VIS) spectrophotometry and potassium dichromate digestion [33]. The concentration of AN was determined by automatic pumping into an AA3 Autoanalyzer (Bran + Luebbe, GmbH, Norderstedt, Germany) with the use of the flow injection analysis technique [31]. The TN and TP concentrations of the samples were determined by UV-VIS spectrophotometry with alkaline potassium persulfate digestion and the Mo-Sb colorimetric method with potassium persulphate digestion, respectively [33]. In addition, the pH of the sample was measured using a portable multiparameter water quality analyzer (Hach, Loveland, CO, USA).

### 2.4. Statistical Analysis

All of the variables were described by the mean ± standard deviation (SD). One-way analysis of variance (ANOVA) was used to compare the removal efficiency of the TIEPD under different rural life events and precipitation intensities. Two-way ANOVAs were used to analyze the effects of the precipitation intensity and rural life events on the N and P removal efficiencies in the ditches and to analyze whether there was an interaction between them, according to least significant difference (LSD) tests at *p* < 0.05. All of the statistical analyses were carried out in SPSS statistical software (IBM SPSS Statistics 22.0). Origin was used to visualize the data through appropriate diagnostic plots.

The removal efficiencies of COD, AN, TN and TP were calculated using the following equation [34]:

(1) $$R=\frac{C_{\mathrm{inlet}}-C_{\mathrm{outlet}}}{C_{\mathrm{inlet}}}\times 100$$

where R is the removal efficiency, %, and $C_{\mathrm{inlet}}$ and $C_{\mathrm{Outlet}}$ are the concentrations of COD, AN, TN and TP at the inlet and outlet sampling points, respectively, mg/L.

## 3. Results

### 3.1. Characteristics of Domestic Sewage Discharge Loads

The average concentrations of COD, AN, TN and TP in the domestic sewage in this study were much higher than those in the Environmental Quality Standard for Surface Water (GB3838-2002) of China for agricultural water use areas and general landscape waters (class V). The discharge volume and pollutant concentration of domestic sewage in villages and towns in mountainous areas fluctuated greatly and were extremely unstable (Table 1). If sewage is discharged directly into natural water bodies, it may seriously affect water environment safety.

The concentrations of COD, AN, TN and TP on nonfair activity days are all lower than those on fair activity days. In contrast, the one-way ANOVA showed that there were no significant differences between fair activity and nonfair activity days in the inlet concentrations of AN, TN and TP (*p* > 0.05). However, the difference in the inlet concentrations of COD was significant (*p* < 0.05) (Table 1).

Light rain days had the highest inlet concentrations of AN and TN, followed by the moderate rain days, while heavy rain days had the lowest inlet concentrations (Table 1). The inlet concentrations of AN and TN on light rain days were significantly different from the concentrations on light rain days and moderate rain days (*p* < 0.05). There were no significant differences in the inlet concentrations of AN and TN between light rain days and moderate rain days (*p* > 0.05). The inlet concentration value of TP on heavy rain days was the highest, followed by light rain days, while moderate rain days had the lowest inlet concentrations. However, there was no significant difference between the different precipitation intensity conditions (*p* > 0.05). Furthermore, the inlet concentration values of COD and pH were the highest on moderate rain days. There was a significant difference in the inlet concentration of COD between moderate rain days and light rain days (*p* < 0.05), but the difference between them and heavy rain days was not significant (*p* > 0.05). There was no significant difference between the different precipitation intensity conditions in the inlet pH value (*p* > 0.05).

### 3.2. Purification Performance of the TIEPD

**Figure 2:**
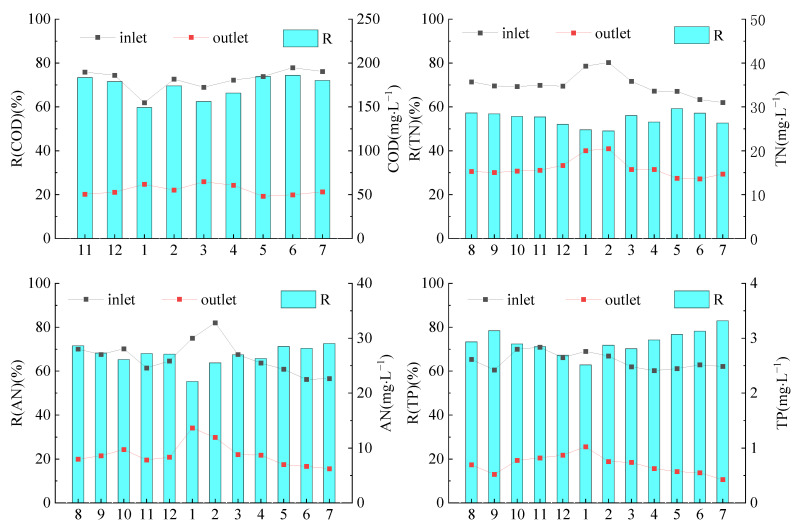
Removal capacity of the TIEPD. Note: Inlet: inlet concentration; Outlet: outlet concentration; R: removal efficiency. The x-axis represents different months (observed from August 2018 to July 2019 except for COD, which was observed from November 2018 to July 2019). The same notation is used in subsequent figures and tables.

In the TIEPD, the average outlet concentrations of COD, AN, TN and TP were 55.21 ± 5.67 mg/L, 8.79 ± 2.17 mg/L, 16 ± 2.23 mg/L and 0.7 ± 0.17 mg/L, respectively (Figure 2). The concentrations all met the level class 1 B standard of the Discharge Standard of Pollutants for Municipal Wastewater Treatment plant (GB 18918-2002) of China. The average removal efficiencies of COD, AN, TN and TP were 69.25 ± 5.01%, 67.23 ± 4.84%, 54.48 ± 3.12% and 73.26 ± 5.6%, respectively.

### 3.3. Effect of Different Loads on the Purification Performance of TIEPD

**Figure 3:**
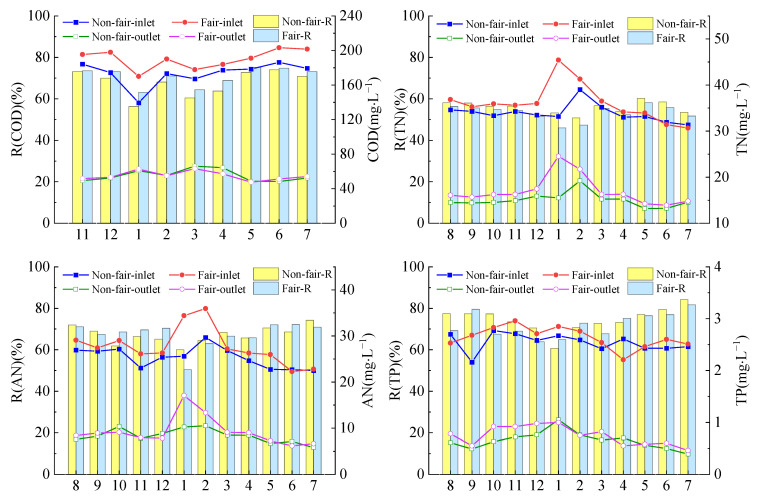
Removal capacity of the TIEPD under different rural life events.

On different rural life event days, the average concentration of the pollutants in the outlet on fair activity days was higher than that on nonfair activity days, except for COD (Figure 3). The concentrations of COD, AN, TN and TP in the outlet on fair activity days and nonfair activity days all met the requirements of the class 1 B standard in the Discharge Standard of Pollutants for Urban Sewage Treatment Plants (GB 18918-2002) (DSP) of China. The removal efficiencies of COD, AN, TN and TP of the TIEPD on the nonfair activity days were 67.7 ± 5.85%, 67.16 ± 3.92%, 55.68 ± 2.74% and 74.48 ± 5.59%, respectively, and were 70.8 ± 4.17%, 67.3 ± 5.75%, 53.27 ± 3.5% and 72.04 ± 5.6%, respectively, on fair activity days. However, there were no significant differences between the concentrations and removal efficiency of COD, AN, TN and TP under different rural life events (*p* > 0.05).

**Figure 4:**
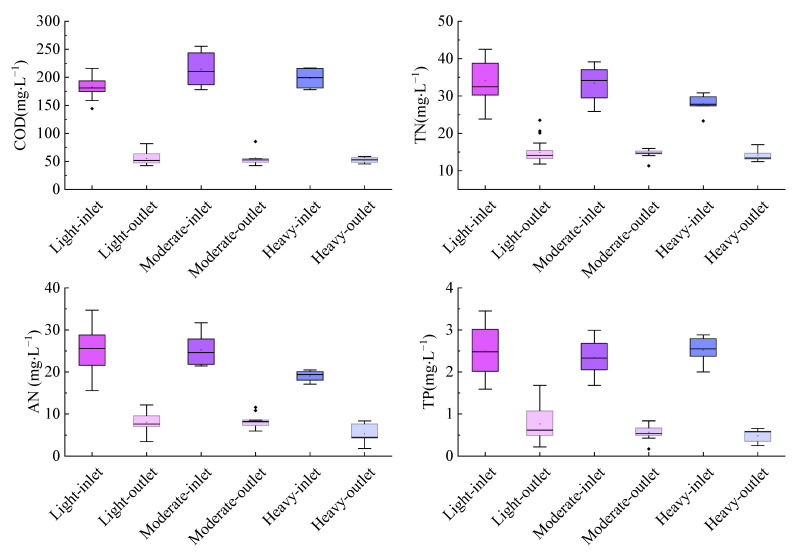
Removal capacity of the TIEPD under different precipitation intensities.

Under different precipitation intensities, the pollutant concentrations in the outlet were significantly lower than those in the inlet (Figure 4). The average outlet concentrations of COD, AN, TN and TP under different precipitation intensities all met the requirements of the class 1 B standard in the DSP of China. One-way ANOVA showed that there were no significant differences between the concentrations of COD, TN and TP under different rural life events (*p* > 0.05). The concentration of AN on heavy rain days was significantly different from that on light rain and moderate rain days (*p* < 0.05). On the light rain days, the average removal efficiencies of COD, AN, TN and TP were 69.84 ± 6.58%, 68.68 ± 6.71%, 56.3 ± 7.41% and 69.31 ± 11.11%, respectively. On moderate rain days, the average removal efficiencies of these indices were 73.07 ± 4.48%, 66.91 ± 6%, 56.27 ± 5.23%, 75.77 ± 8.93%, respectively. Additionally, they were 73.94 ± 2.54%, 69.85 ± 12.16%, 48.41 ± 10.68%, 79.43 ± 6.04%, respectively, on heavy rain days. Moreover, the differences in the removal efficiencies of COD, AN, TN and TP between different precipitation intensities were not significant (*p* > 0.05).

The two-way ANOVAs suggested that the precipitation intensity and the interaction between the precipitation intensity and rural life events had significant influences on the COD removal efficiency (*p* < 0.05) (Table 2). In addition, rural life events had a significant effect on the removal efficiency of AN (*p* < 0.05). Beyond that, the precipitation intensity, rural life events and their interaction had no significant influences on the removal efficiencies of COD, AN, TN and TP (*p* > 0.05).

## 4. Discussion

### 4.1. Effect of Different Loads on the Purification Performance of the TIEPD

The water quality was weakly alkaline, and the concentrations of nitrogen, phosphorus and organic matter in the water seriously exceeded the standard. Thus, it was necessary to treat the sewage before it was discharged. Untreated domestic sewage and storm runoff from roads are the main water sources for residential ditches [8,35]. Under different rural life event conditions, domestic sewage discharge was evidently different due to various reasons, such as commodity trading, small gatherings, increased flow of villagers, increased water consumption in hotels and increased wastewater in slaughterhouses. Under different precipitation intensity conditions, the living activities of residents also change, resulting in differences in sewage discharge. In addition, in the precipitation runoff, the concentration of the pollutants is gradually diluted, and the washout effect of the rainwater appears in continuous precipitation [36,37].

From the analysis of the removal mechanism of the overall removal efficiency of the TIEPD, the removal mechanisms of various pollutants were not the same. COD represents the concentration of organic pollutants in domestic sewage. Under anaerobic conditions, organic compounds are transformed into volatile fatty acids (VFAs) and other low-molecular-weight compounds [38], the latter of which are then broken down into methane and carbon dioxide. More than 50% of COD in the sewage was removed in this way. In this study, sufficient dissolved oxygen (DO) was obtained to breakdown COD by means of three-stage waterfall aeration. The TIEPD was also the habitat for various microorganisms, which undertook the predominant function of degrading the organic pollutants [39,40]. Residual COD was further reduced by microbial decomposition and filter interception [28]. Nitrogen removal is the result of the joint action of plants and microorganisms [29,41]. First, all kinds of plants in the TIEPD need to absorb part of the inorganic nitrogen (NH_4_^+^ and NO_3_^−^) as a nutrient source in the growth and reproduction process, but the domestic sewage nitrogen concentration is high; plant absorption only accounts for a small part, and the remaining nitrogen is mainly purified by microbial nitrification and denitrification [42,43]. NH_4_^+^-N is mostly transformed into NO_3_^−^-N under the action of nitrifying bacteria and nitrobacteria. Finally, NO_3_^−^-N is transformed into N_2_ and N_2_O through denitrification and released into the atmosphere [44,45]. The TIEPD consists of three stages, each of which includes an amphibious, a plant reaction pond and an aquatic plant reaction pond, so the plant absorption, aerobic nitrification and anaerobic denitrification process can be recycled, and the nitrogen removal efficiency is better. Phosphorus exists in the forms of inorganic phosphate, granular phosphorus and dissolved organic phosphorus in sewage. Phosphorus is primarily removed in the TIEPD by plant absorption, physical filtration and microbial action [28,29]. Granular phosphorus mainly adheres to suspended solids and is removed due to sedimentation [46,47]. Microorganisms mainly remove dissolved organophosphorus [25], and phosphor-accumulating bacteria remove inorganic phosphate [48]. Finally, phosphorus salts are removed through sludge discharge.

The removal efficiencies of TN and TP by the TIEPD were 24.48% and 29.26% higher than those of the constructed wetland (CW) in Korea [23]. In addition, the COD removal efficiency of the TIEPD is 19.25% higher than that of biological ecological processes for rural domestic sewage treatment in the Taihu region of China [28]. Furthermore, the removal efficiencies were 48% and 61% for TN and TP in the vegetated ditches in the hilly area of Southwest China and 51% for TN in the hilly area of Central China [49,50].The TIEPD showed better performance in the treatment of domestic sewage in rural areas. In addition, the construction and operation cost of sewage treatment facilities in rural areas is an important factor affecting their promotion [9]. According to the sampling calculation method of taxable pollutant emissions of the environmental protection tax in Sichuan Province, in the case of direct discharge of domestic sewage, the owner of the sewage discharge pays a pollution tax of 0.39 USD/ton. The construction cost of the TIEPD is approximately 49644 USD, and the maintenance and operation cost is approximately 0.01 USD/ton, which is far lower than that of other ecological purification systems [51]. Thus, it is evident that, the TIEPD has better economic benefits. Furthermore, the study area is located in the hills of central Sichuan, China, with less available land and scattered residents. The TIEPD, as a relatively perfect rural decentralized domestic sewage treatment technology, has a small land demand and is more practical for purifying domestic sewage in mountainous villages and towns. At the same time, some environmental benefits can also be realized when treated sewage is used for farmland irrigation.

### 4.2. Effect Factors of the Purification Performance of the TIEPD

On fair activity days, the concentrations of COD, AN, TN and TP in the sewage increased, and the nutrients in the water increased, leading to the proliferation of some heterotrophic bacteria, improving the activity of heterotrophic bacteria, and increasing the purification efficiency of COD [29]. However, with the increasing concentration of various pollutants, other microbial communities are destroyed, and the survival conditions of the microorganisms are limited, so their growth is affected, which has varying degrees of influence on nitrification and denitrification reactions and phosphorus removal reactions. Heterotrophic nitrification and aerobic denitrification (HN-AD) promote the removal of N. The nitrogen source is one of the factors that may affect the performance of HN-AD bacteria [42,43]. In addition, the greater the flow rate or influent N concentration, the lower the N removal efficiency [34]. Therefore, the removal efficiencies of TN and AN on fair activity days were lower than those on nonfair activity days. TP removal was positively correlated with algae treatment and different HRTs. Algae would improve TP removal and changing the HRT value would also improve the removal. In addition, the lower N/P ratios indicated a stronger synergistic effect between phosphorus and nitrogen removal [52]. Thus, TP removal showed a tendency to fluctuate with different human activities.

The purification efficiencies of COD, AN, TN and TP were significantly different under different precipitation intensities. On light rain days, due to the low precipitation intensity, there is no surface runoff on the ground, so the influent concentration and purification efficiency of all pollutants are almost the same as normal. During moderate rain days and heavy rain days, surface runoff with fast velocity is generated on the ground, which results in N, P and other pollutants originally existing on the ground and roof flowing into the TIEPD with surface runoff [28], leading to a large difference in the concentration of each pollutant in the water and a change in the purification efficiency. Precipitation can increase the oxygen content in water and improve microbial life activities, thus promoting COD and TP removal in wastewater [29,53]. It could also promote nitration, improving the removal efficiency of AN [29]. In addition, an increase in the oxygen content in water is not conducive to denitrification [29]. Thus, NO_3_^−^-N could accumulate in sewage, decreasing the removal efficiency of TN.

The change in rural life events has little effect on the removal efficiency of COD, which is roughly similar to the research results of Liang Kang et al. [54]. In this study, the precipitation intensity and the interaction between rural life events and the precipitation intensity had a significant influence on the COD removal efficiency (*p* < 0.05). This is because microbial action has an important effect on COD removal [29]. In addition, rural life events had a significant effect on the removal of AN in the TIEPD (*p* < 0.05). Indeed, the concentration of AN has a significant effect on the efficiency of AN removal [34]. Furthermore, the removal of TN and TP by the TIEPD was less affected by rural life events, the precipitation intensity and their interaction (*p* > 0.05), which meant that the TIEPD exhibited a good stability for the removal of pollutants.

### 4.3. Implications for TIEPD Management

In this study, the TIEPD showed a good removal potential towards pollutants (Figure 2). However, management still needs to be strengthened to solve the problem of a reduced removal efficiency of the system under conditions such as mixed rain and sewage (Figure 3 and Figure 4). The long-term operation of the TIEPD still faces a high risk of failure if it is not well maintained [9]. Thus, measures such as timely dredging, medium renewal and plant harvesting ensure the long-term sustainable operation of the system [55]. Xia et al. found that the capacity to remove TN and TP using several harvestings of aboveground plant tissue is higher than the capacity of annual harvesting [56]. The study by Matt et al. showed significant differences in the microbial activity and pollutant concentrations at the outlet between pre-dredged and post-dredged treatment systems [55]. At the same time, we should make rational use of natural ditches in mountainous areas and transform or implement [8] combined control measures such as rainwater and sewage diversion, which can also further improve the purification capacity of the TIEPD for domestic sewage in villages.

This study shows the effectiveness of the TIEPD as a nondynamic and three-dimensional ecological purification technology in rural areas in mountainous areas of the upper reaches of the Yangtze River and provides certain technical support for fundamentally solving rural, decentralized domestic sewage in the upper reaches of the Yangtze River. In addition, this study also provides a scientific basis for reducing rural wastewater discharges in mountainous and hilly areas of subtropical countries and developing countries.

## 5. Conclusions

This study has proven that the TIEPD runs stably during the working period and has good stability and load resistance with no large failures under different rural life events and precipitation intensities.

From the perspective of the removal efficiency, the average purification efficiencies of COD, AN, TN and TP of the TIEPD were above 50% during the working period. The average removal efficiencies of COD, AN, TN and TP were 69.25%, 67.23%, 54.48% and 73.26%, respectively. The average effluent concentration of each pollutant can meet the class 1 B standard of the DSP of China.

Under different load concentrations, the effluent concentrations of COD, AN, TN and TP can still meet the A or B standards of the DSP of China. The system has good stability in removing pollutants.

The TIEPD has good ecological and economic benefits. Therefore, the TIEPD, as a relatively perfect rural decentralized domestic sewage treatment technology, can be constructed and operated in similar areas at a low cost, which has a certain promotion value for the treatment of domestic sewage in the upper reaches of the Yangtze River and even in mountainous and hilly areas of subtropical and developing countries.

However, the removal of pollutants in the TIEPD may also be influenced by other factors. It is necessary to conduct long-term hydraulic experiments to further understand the mechanism influencing the RNPS removal efficiency of the TIEPD.

## Figures and Tables

**Table 1:** Discharge characteristics of domestic sewage.

	Annual Average Discharge	Rural Life Events		Precipitation Intensities		
		Nonfair	Fair	Light	Moderate	Heavy
COD (mg/L)	181.69 ± 11.80	173.27 ± 13.12 a	190.10 ± 10.48 b	182.56 ± 17.33 b	214.22 ± 28.77 a	198.42 ± 17.55 ab
AN (mg/L)	26.53 ± 3.02	25.33 ± 2.17 a	27.73 ± 3.87 a	25.59 ± 4.67 a	25.25 ± 3.44 a	19.00 ± 1.27 b
TN (mg/L)	35.00 ± 2.84	33.90 ± 1.86 a	36.09 ± 3.82 a	34.09 ± 5.25 a	33.41 ± 4.19 a	27.83 ± 2.59 b
TP (mg/L)	2.59 ± 0.18	2.54 ± 0.16 a	2.63 ± 0.20 a	2.50 ± 0.52 a	2.33 ± 0.40 a	2.52 ± 0.32 a
pH	7.42 ± 0.17	7.42 ± 0.25 a	7.26 ± 0.22 a	7.34 ± 0.28 a	7.37 ± 0.20 a	7.27 ± 0.13 a

Note: Different lowercase letters (a, b and ab) in the same row indicate significant differences among effluents at the 0.05 level. The same notation is used in subsequent figures and tables.

**Table 2:** Influence of pollutant load on removal efficiency of the TIEPD.

	Precipitation Intensities			Rural Life Events			Precipitation Intensities * Rural Life Events		
	F	P	df	F	P	df	F	P	df
TN	0.053	0.948	2	0.972	0.334	1	0.978	0.391	2
AN	0.993	0.385	2	4.503	0.044	1	1.924	0.168	2
TP	0.407	0.529	2	1.081	0.355	1	0.066	0.936	2
COD	5.28	0.035	2	1.576	0.236	1	4.605	0.025	2

Note: F: F-statistic (the statistic that fits the F distribution under the null hypothesis); P: *p* value (the probability that an outcome more extreme than the sample observation obtained will occur when the original hypothesis is true); df: degrees of freedom; Precipitation Intensities * Rural Life Events: the interaction of precipitation intensities and rural life events.

## Data Availability

Not applicable.
